# ﻿*Gymnostachyumcalcicola* (Acanthaceae), a new species from limestone karst of Peninsular Malaysia

**DOI:** 10.3897/phytokeys.242.122869

**Published:** 2024-06-03

**Authors:** Abdul Rahman Rafidah, Abdul Rahman Ummul Nazrah, Poh Teck Ong

**Affiliations:** 1 Forest Research Institute Malaysia, 52109 Kepong, Selangor, Malaysia Forest Research Institute Malaysia Selangor Malaysia

**Keywords:** Andrographideae, endemic, flora, Kelantan, taxonomy

## Abstract

A new species, *Gymnostachyumcalcicola* Rafidah, **sp. nov.** (Acanthaceae) is described from limestone karst in Peninsular Malaysia. Characters distinguishing it from related species, colour photographs, botanical illustration and provisional conservation status are provided.

## ﻿Introduction

*Gymnostachyum* Nees (Acanthaceae) is a genus consisting of about 30–50 species distributed mainly in tropical Asia (Hu et al. 2011; Deng 2014; Mabberley 2017; Deng et al. 2020; Manzitto-Tripp et al. 2022). The genus is characterized by a combination of two stamens and many seeds (Hansen 1985), absence of staminodes, ovary with 3-several ovules per locule, capsule cylindrical, and seeds compressed, covered with hygroscopic hairs (Lindau 1895). It belongs to tribe Andrographideae (Lindau 1895; Scotland 1992; Scotland and Vollesen 2000; McDade et al. 2008), however Somprasong et al. (2015) placed *Gymnostachyum* under subtribe Andrographinae. In Peninsular Malaysia, Clarke (1908) recognized eleven species of *Gymnostachyum*, including ten new species, while Ridley (1923) recorded *Gymnostachyum* with 13 species (including four doubtful species) and described a new species *Gymnostachyumhirtum* Ridl. from Perak (Ridley 1926). Tan et al. (2014) described *Gymnostachyumkanthanense* Kiew from a limestone hill, Gunung Kanthan, Perak.

The discovery of this distinctive new species is part of the comprehensive survey of the Peninsular Malaysia limestone flora in 2017. The species was found on the limestone hills in Federal Land and Development Authority (FELDA) Chiku and Perasu in Kelantan, and is endemic in Kelantan. The species inhabits partially shaded areas, and roots in cracks with thin soil layer on rocks at the base to the summit of karst limestone. After carefully examining the herbarium specimens and living material and reviewing the relevant literature, here we concluded that the newly discovered plants represented an undescribed species. Herewith, the new species is described and illustrated in detail.

## ﻿Materials and methods

Measurements and morphological character assessments of the new species were undertaken and described using specimens collected from their natural habitat and living materials grown in the Forest Research Institute Malaysia (FRIM) Biodiversity Nursery. Photographs of the habit and all parts of the plants were made and botanical line drawings of floral and fruiting parts were prepared. Materials collected were deposited at the herbarium of Forest Research Institute Malaysia (KEP) and the flowers were preserved into the spirit collection in Copenhagen mixture. The available *Gymnostachyum* specimens from other herbaria such as BK, BKF, CAL, KLU, SING, UKMB and PSU were examined (herbarium acronyms follow Thiers 2024, continuously updated). In addition, images of the type specimens of *Gymnostachyum* were obtained from JSTOR Global Plants (http://plants.jstor.org) and Tropicos (http://www.tropicos.org). The provisional conservation assessment is based on the IUCN Red List Categories and Criteria Version 3.1 (IUCN 2012) and guidelines of the IUCN (2022).

## ﻿Taxonomic treatment

### ﻿ Gymnostachyum calcicola

Taxon classificationPlantaeLamialesAcanthaceae

Rafidah sp. nov.

1272C8CB-5D80-5AF3-AC69-8F6EB9195EAD

urn:lsid:ipni.org:names:77342878-1

Figs 1 , 2 , 3

#### Diagnosis.

Unique among Peninsular Malaysian species of *Gymnostachyum* by having a racemose inflorescences occasionally lower axils with pairs of flowers and dichasium inflorescence with opposite branches. *Gymnostachyumcalcicola* shows affinity with Gymnostachyumdecurrensvar.decurrens and var. robinsonii by its crowded rosette leaves at the base, however it is different in the inflorescences type.

#### Type.

Peninsular Malaysia. Kelantan: Chiku, FELDA Chiku, 11 October 2017, *Aliaa-Athirah et al. FRI 90707* (holotype KEP, isotype SING).

**Map 1.**
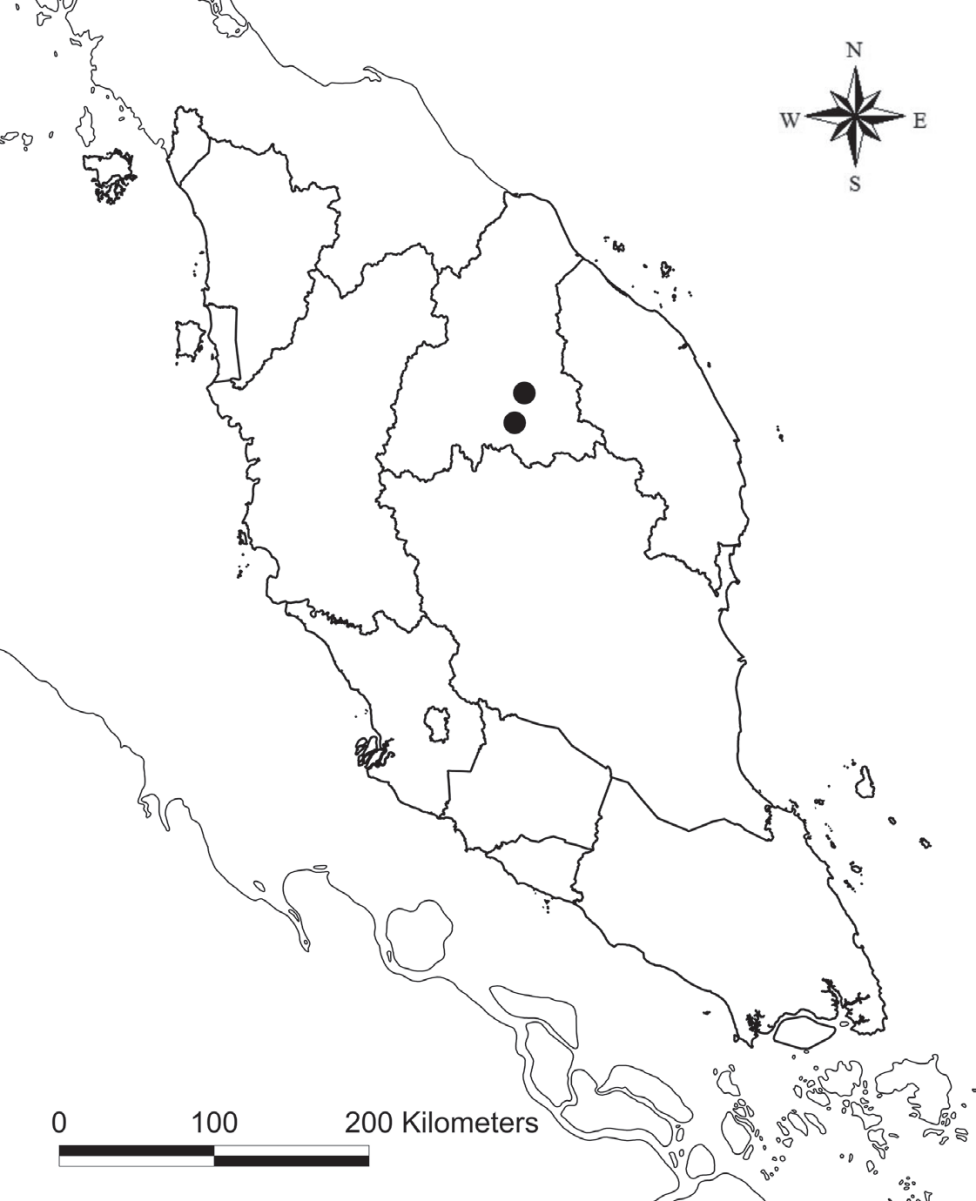
Map of the Peninsular Malaysia indicating the location of *Gymnostachyumcalcicola* in Peninsular Malaysia.

#### Description.

Rosette herbs. Leaves opposite; petiole pale green, to 7 cm long, not winged; lamina membranous to subcoriaceous, dark green above, sometimes with grey blotches, white-green beneath, narrowly oblong to elliptic, ca. 11 × 4–5 cm, base abruptly truncate, decurrent for 2 cm, margin entire, slightly wavy, apex unequal, slightly acute to blunt; midrib and veins slightly sunken above, glabrous beneath; lateral veins ca. 9–10 pairs. Inflorescences green, erect, terminal, racemose occasionally lower axils with pairs of flowers, dichasium with opposite branches, rachis up to 30 cm long or more, minutely hairy, branches ca. 14 cm long with up to 5 flowers; bracts green, ca. 1 mm long; pedicels very short, ca. 0.5 mm long; bracteoles minute, 0.5 mm long. Flowers suberect. Calyx divided near the base, lobes 5, equal, narrowly linear, 2–3 × 0.5–1 mm long, green, hairy outside, clasping the corolla tube. Corolla white outside, inside white with scattered minute purple spots except for the deep purple lower lip, deep purple at the median lobe, minutely glandular hairy outside, ca. 12–14 mm long, narrowly cylindric at base, tube 10 × 1 mm, expanding distally to funnel-shaped throat, upper lip erect, ca. 3 mm long, apex slightly bilobed, flat, lower lip unequal, the middle lobe much shorter. Stamens 2, filaments white, 6–7 mm long, inserted at the base of the throat, glabrous, anthers purple-white, inserted, positioned below the apex of upper lip, narrowly oblong, ca. 2 mm long, thecae 2, equal, both with minute mucronate appendages at base, densely covered with short stalked glandular hairs with dense white hairs along longitudinal line of dehiscence; staminodes absent. Nectary annular, cream-coloured, ca. 1 mm high, upper margin entire. Ovary green, cylindric, 1–1.5 mm long, densely covered in short glandular hairs, ovules many per locule; style white, up to 8 mm long, sparsely hairy, stigma hooked, less than 1 mm long, positioned between the anthers. Capsule narrowly cylindric, 10–12 × 1–2 mm, retinacula 6–7 per locule, calyx persisting after the fruit has fallen. Seeds up to 12 (probably more), obliquely ovoid, strongly compressed, ca. 1 mm longitudinal, surface minutely pitted, hairy.

**Figure 1.**
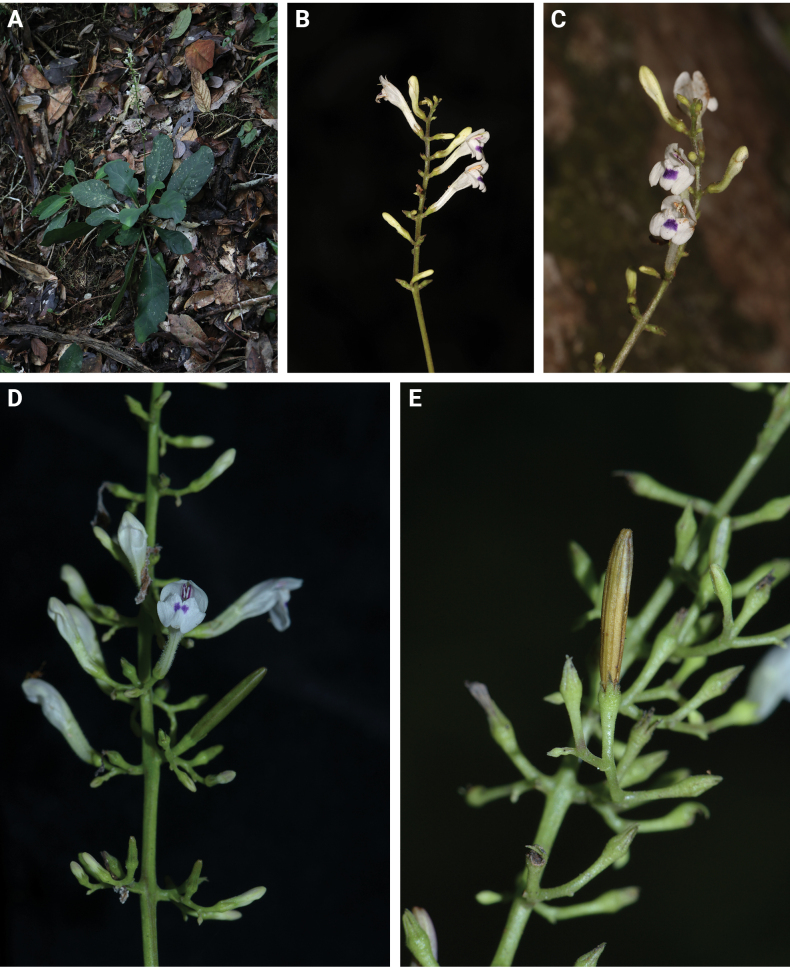
*Gymnostachyumcalcicola* Rafidah **A** habit **B** flower (side view) **C** flower (front view) **D** portion of inflorescence showing dichasial cymose branches **E** portion of infructescence.

#### Distribution and habitat.

Endemic to Kelantan, Peninsular Malaysia; known only from limestone hills. Species grows in small populations, always observed in shaded areas, rooting in cracks on thin soil on limestone rocks, almost to the summit of the hill.

**Figure 2.**
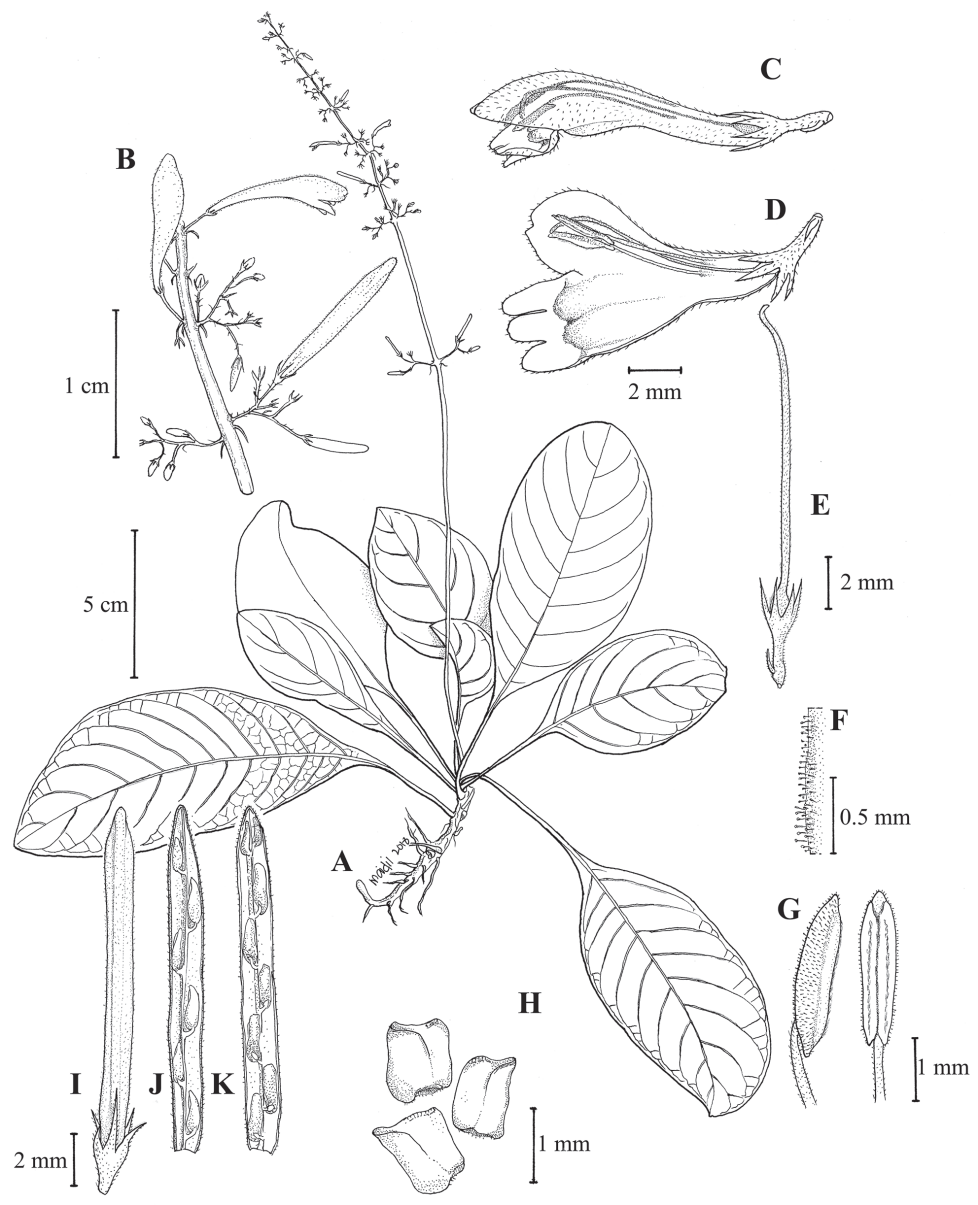
*Gymnostachyumcalcicola* Rafidah **A** flowering plant **B** portion of inflorescence with flowers **C** flower **D** flower with opened corolla **E** bract, calyx and carpel with corolla and stamens removed **F** indumentum of anther along longitudinal line of dehiscence **G** anthers (dorsal and ventral views) **H** seeds **I** fruit **J-K** attachment of seeds (all drawn by Mohd Aidil Nordin).

#### Etymology.

The specific epithet refers to the limestone habitat of this species.

#### Provisional IUCN regional conservation status.

Endangered B2 ab(i,ii,iii). Most Chiku limestone hills were visited, but only two to three hills were home to this new species. A part of Chiku limestone hills is scheduled for quarries. The survival of the species remains uncertain. The species also has been recorded in Perasu limestone hills, about 40 km away from Chiku hills. Parts of FELDA Perasu limestone have been quarried, and surrounding areas are currently disturbed by the road constructions. All hills lie outside the network of Protected Areas.

**Figure 3.**
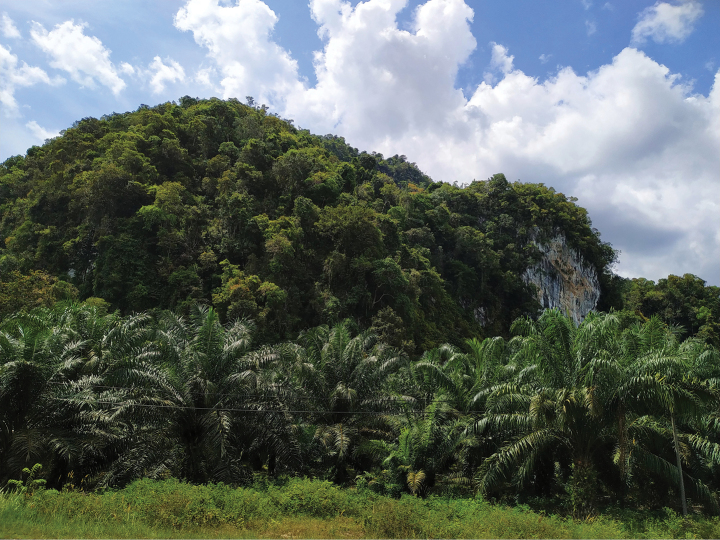
Home of *Gymnostachyumcalcicola*.

#### Additional specimens examined.

Peninsular Malaysia, Kelantan: FELDA Chiku, 11 October 2017, Aliaa-Athirah *et al*. 90712 (KEP), 10 October 2017, Wan Syafiq *et al*. FRI 90123 (KEP); FELDA Perasu, 24 April 2019, Rafidah FRI 93064 (KEP).

#### Notes.

Ridley (1923) divided the Peninsular Malaysian species into two groups: (i) dwarf plants with single flower of long inflorescences and; (ii) shrubby plants with elongate stem. This new species falls into the first group (Table 1) together with GymnostachyumdecurrensStapfvar.decurrens and var. robinsonii (Ridl.) J.B. Imlay, *G.diversifolium* C.B.Clarke, *G.pallens* C.B.Clarke, *G.kanthanense*, and doubtful species recorded for Peninsular Malaysia, *G.ceylanicum* Arn. & Nees. The new species shows affinity with Gymnostachyumdecurrensvar.decurrens and var. robinsonii by its crowded rosette leaves at the base and its racemose and spicate inflorescences and *G.pallens* from its branched peduncle. It differs from the other species by not having a winged petiole and in having racemose inflorescences, occasionally lower axils with pairs of flowers and dichasium inflorescence with opposite branches.

**Table 1.** Comparison of Peninsular Malaysia species in *Gymnostachyum* in Group 1.

Characters / Species	*G.calcicola*	G.decurrensvar.decurrens	G.decurrensvar.robinsonii	*G.diversifolium*	G.ceylanicum*	*G.kanthanense*	*G.pallens*
Petiole	not winged	broadly-winged	winged	winged	winged	not winged	winged
Lamina							
Base	abruptly truncate	broad	abruptly truncate	broad	unknown	abruptly rounded or truncate	abruptly truncate
	decurrent for 2 cm	undulate margin, decurrent	decurrent	decurrent	undulate margin, decurrent	decurrent for 2–8 mm	shortly decurrent
Indumentum surface (both)	Glabrous	pubescent	scabrid	pubescent	pubescent	pubescent	densely punctate
Inflorescence							
Type	racemose	spikes	racemes	spikes	spikes	spikes	spike
	dichasium inflorescence with opposite branches	several	branched	1 to 3	several	3	slightly branched
Flower	occasionally lower axils with pairs of flowers	single	single	single	single opposite or in long spikes in short cymes	on one side of the rachis	single
Calyx							
Colour	green	purple	green	pale green	dark green	purplish green	green
Corolla							
colour	white	white	unknown	pale blue	pinkish / pale purple	white tinged purple	white
length (mm)	12–14	12–15	10	25	12	18	12
lower lip (median)	deep purple	violet	unknown	purplish (all)	yellow	deep purple	white
indumentum	minutely glandular hairy	glandular pubescent	glandular pubescent	glandular and e-glandular hairs	unknown	stalked glandular hairs	unknown
Capsule							
length (mm)	10–12	18	12	unknown	unknown	12	> 12

*Gymnostachyumceylanicum absolutely indistinguishable from Sri Lanka specimen from the corolla colour.

## Supplementary Material

XML Treatment for Gymnostachyum calcicola

## ﻿Additional information

### Author ORCIDs

Abdul Rahman Rafidah https://orcid.org/0000-0003-1055-4894

Abdul Rahman Ummul Nazrah https://orcid.org/0009-0002-7284-2592

### Data availability

All of the data that support the findings of this study are available in the main text.
